# Gene delivery to pancreatic exocrine cells *in vivo* and *in vitro*

**DOI:** 10.1186/1472-6750-12-74

**Published:** 2012-10-22

**Authors:** Isabelle Houbracken, Luc Baeyens, Philippe Ravassard, Harry Heimberg, Luc Bouwens

**Affiliations:** 1 Cell Differentiation Lab, Diabetes Research Center, Vrije Universiteit Brussel, Laarbeeklaan 103, Brussels, B-1090, Belgium; 2 Beta Cell Neogenesis Lab, Diabetes Research Center, Vrije Universiteit Brussel, Laarbeeklaan 103, Brussels, B-1090, Belgium; 3 Université Pierre et Marie Curie-Paris 6, Biotechnology & Biotherapy Team, Centre de Recherche de l’Institut du Cerveau et de la Moelle épinière (CRICM), UMRS 975, Paris, 75013, France; 4 CNRS, UMR 7225, Paris, 75013, France; 5 Inserm, U 975, Paris, 75013, France

**Keywords:** Lentiviral vector, Adenoviral vector, Lipofection, Gene transfer, Pancreas, Acinar cell

## Abstract

**Background:**

Effective gene transfer to the pancreas or to pancreatic cells has remained elusive although it is essential for studies of genetic lineage tracing and modulation of gene expression. Different transduction methods and viral vectors were tested in vitro and in vivo, in rat and mouse pancreas.

**Results:**

For in vitro transfection/transduction of rat exocrine cells lipofection reagents, adenoviral vectors, and Mokola- and VSV-G pseudotyped lentiviral vectors were used. For in vivo transduction of mouse and rat pancreas adenoviral vectors and VSV-G lentiviral vectors were injected into the parenchymal tissue. Both lipofection of rat exocrine cell cultures and transduction with Mokola pseudotyped lentiviral vectors were inefficient and resulted in less than 4% EGFP expressing cells. Adenoviral transduction was highly efficient but its usefulness for gene delivery to rat exocrine cells in vitro was hampered by a drastic increase in cell death. In vitro transduction of rat exocrine cells was most optimal with VSV-G pseudotyped lentiviral vectors, with stable transgene expression, no significant effect on cell survival and about 40% transduced cells. In vivo, pancreatic cells could not be transduced by intra-parenchymal administration of lentiviral vectors in mouse and rat pancreas. However, a high efficiency could be obtained by adenoviral vectors, resulting in transient transduction of mainly exocrine acinar cells. Injection in immune-deficient animals diminished leukocyte infiltration and prolonged transgene expression.

**Conclusions:**

In summary, our study remarkably demonstrates that transduction of pancreatic exocrine cells requires lentiviral vectors in vitro but adenoviral vectors in vivo.

## Background

The pancreas is a mixed gland with an exocrine and an endocrine component. The exocrine portion makes up more than 95% of the volume of the pancreas, the endocrine portion 1–2% [1]. The exocrine pancreas consists of duct, centroacinar and acinar cells, the latter being the most abundant cell type. Acinar cells produce digestive enzymes which they secrete into the duodenum via ducts. In 2 major pancreatic pathologies, pancreatitis and pancreas cancer, the acinar cells are replaced by duct cells, a process termed acinoductal metaplasia [2]. Pancreas cancer has a dismal prognosis: the overall 5-year survival rate among patients with pancreas cancer is less than 6% [3]. Exocrine tumours are the most common form of pancreas cancer from which 95% are pancreatic ductal adenocarcinomas (PDAC). PDAC is thought to arise from ductal precursor lesions, including pancreatic intraepithelial neoplasias (PanIN), which accumulate mutations and become progressively dysplastic, finally forming metastatic tumours [4]. Many recent studies in mice indicate by genetic lineage tracing that at least a part of PDAC and PanIN arise from acinar cells that are reprogrammed into a preneoplastic state [4-8]. Lineage tracing also revealed acinar-to-ductal transdifferentiation, both in caerulein-induced pancreatitis and following pancreatic transforming growth factor alpha overexpression in mice [9,10]. In addition, cultured acinar cells from mice [11,12] and humans [13] can transdifferentiate to duct cells.

Also in the context of diabetes mellitus the acinar cells represent an interesting cell population of the pancreas. Diabetes is a group of metabolic diseases characterized by high blood glucose levels which result from defects in insulin secretion, or action, or both. It is a major and growing public health problem throughout the world. Beta cell transplantation can restore the functional beta cell mass in diabetic patients but it is seriously hampered by donor shortage. This problem could be solved by generating more beta cells by reprogramming more readily available cell types unaffected by the disease. Acinar cells, the most abundant cell type in the pancreas, represent an attractive source for beta cell replacement therapy. Lineage tracing studies in vitro as well as in vivo have clearly shown that rodent acinar cells can be converted into functional beta cells [14-17]. The ability of acinar cells to become reprogrammed is further supported by acinar transdifferentiation into hepatocytes [18,19] and adipocytes [20]. Their remarkable plasticity and their involvement in 3 major pancreas pathologies make acinar cells a highly interesting cell type. Obviously, an efficient method for gene delivery in pancreatic acinar cells would be a useful tool for genetic lineage tracing, overexpression and knock-down studies, and for gene therapy. Several viral vectors such as adenoviral vectors [13,15,17,21-28], lentiviral vectors [29-34] and adeno-associated viral vectors (AAV) [27,35-37] have been used for gene delivery in pancreas. Although long-term and robust gene transfer in pancreas has already been achieved with AAV vectors and despite several advantages of AAV vectors compared to other vectors such as very low immunogenicity, replication defectiveness, lack of pathogenicity and broad tropism, their major disadvantage of a limited cloning capacity precluded their usage in this study. Because of this characteristic, AAV vectors are less suitable for delivery of larger genes for lineage tracing or overexpression studies. In this comparative study, we opted for lentiviral and adenoviral vectors, next to lipofection. The different transfection/transduction methods were tested in vitro and in vivo in rat and mouse pancreas. We conclude that vesicular stomatitis virus-glycoprotein (VSV-G) pseudotyped lentiviral vectors are the most optimal for in vitro gene transfer, whereas for in vivo transduction of rodent pancreas, adenoviral transduction reached the highest efficiency.

## Results

### In vitro transfection of exocrine pancreatic cells is inefficient

**Figure 1:**
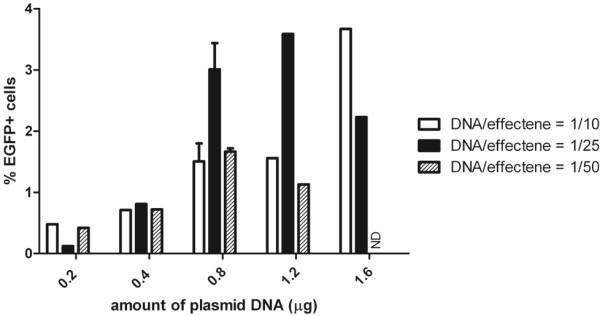
**Transfection of isolated rat exocrine pancreas cells with effectene.** Quantification of the percentage EGFP expressing cells on day 8 in culture after transfection of rat exocrine pancreas on day 1 with pEGFP-N2 and effectene with varying amounts of plasmid DNA and ratios of DNA/effectene.

Primary cultures of exocrine cells isolated from rat pancreas were transfected with 2 different commercial lipofection reagents, lipofectamine 2000 (cationic liposome) and effectene (a two-component non-liposomal lipid formulation). Transfection was performed on the day of isolation with pEGFP-N2 (day 1) and the efficiency was determined by analyzing the percentage of enhanced green fluorescent protein (EGFP)+ cells. While it is reported that cell lines of the exocrine pancreas could be transfected at high yields using lipofectamine 2000 [13,38-41], we found that less than 0.1% of primary cells were transfected and expressed EGFP (not shown). When varying amounts of plasmid DNA and varying ratios of DNA to effectene reagent were used, EGFP+ cells appeared 24 hours after transfection ( Additional file 1: Figure S1) but only ≤ 3.7% of total cells expressed EGFP on day 8 (Figure 1). A similar efficiency was obtained at earlier time points (Additional file 1: Figure S1).

### In vitro transduction of rat exocrine pancreatic cells using mokola and VSV-G pseudotyped lentiviral vectors

**Figure 2:**
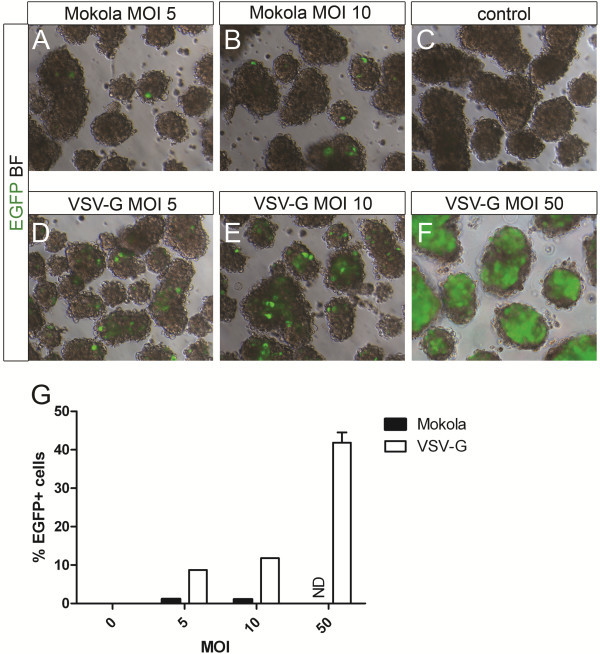
**Transduction of isolated rat exocrine pancreas cells using Mokola and VSV-G pseudotyped lentiviral vectors.** (**A**-**F**) Day 3 in culture of transduced rat exocrine pancreas cells with Le^Mo^CMV-EGFP (**A**) MOI 5, (**B**) MOI 10 or (**D**) Le^VSV-G^CMV-EGFP MOI 5, (**E**) MOI 10, (**F**) MOI 50. (**C**) Untransduced cells showed no fluorescence. The expression of the reporter increased with higher MOI and was better in Le^VSV-G^CMV-EGFP as compared to Le^Mo^CMV-EGFP. Merged pictures of bright field and green fluorescence. (**G**) Quantification of the percentage of EGFP expressing cells on day 8 in culture after transduction with Le^Mo^CMV-EGFP or Le^VSV-G^CMV-EGFP of rat exocrine pancreas on day 1.

The efficiency of Mokola and VSV-G pseudotyped lentiviral vectors was compared in transducing isolated rat exocrine pancreatic cells. Transduction was performed on day 1 (the day of isolation) with a recombinant lentiviral vector that expressed EGFP under the control of the ubiquitous and constitutively active cytomegalovirus (CMV) promoter (LeCMV-EGFP) and whose envelope was pseudotyped with glycoproteins either from Mokola virus (Le^Mo^CMV-EGFP) or from VSV (Le^VSV-G^CMV-EGFP). During culture, EGFP was stably expressed (Figure 2A-F) and on day 8, the fraction of EGFP+ cells was counted at different MOI’s (Figure 2G). The efficiency to transduce isolated rat exocrine cells with Le^VSV-G^CMV-EGFP was 8 to 11 times higher than with Le^Mo^CMV-EGFP at multiplicity of infection (MOI) 5 and MOI 10. Transduction with Le^VSV-G^CMV-EGFP at MOI of 50 resulted in 41.9 ± 2.6% EGFP+ cells on day 8 (Figure 2 F,G).

### Comparison of in vitro adenoviral and lentiviral transduction of rat exocrine cells

**Figure 3:**
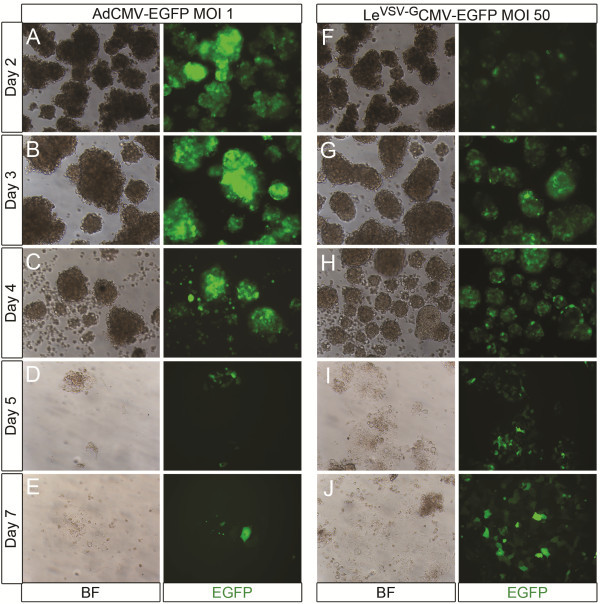
**Culture of AdCMV-EGFP and Le**^**VSV-G**^**CMV-EGFP transduced rat exocrine pancreas cells.** (**A**-**J**) Culture of rat exocrine pancreas cells transduced with (**A**-**E**) AdCMV-EGFP MOI 1 and (**F**-**J**) Le^VSV-G^CMV-EGFP MOI 50, (**A**, **F**) day 2, (**B**, **G**) day 3, (**C**, **H**) day 4, (**D**, **I**) day 5 and (**E**, **J**) day 7 in culture. Adenoviral transduction resulted in higher transduction efficiencies and higher levels of EGFP expression on (**A**) day 2 and (**B**) day 3, when compared to the lentiviral vectors (**F-G**). However, from (**C**) day 4 on, massive cell death occurred in the adenovirally transduced condition and the monolayer formation was disturbed. On the contrary, the cell viability in lentivirally transduced cells remained normal and they had a strong and stable expression of the transgene.

To compare the transduction efficiency of adenoviral (AdCMV-EGFP) or lentiviral (Le^VSV-G^CMV-EGFP) vectors, rat exocrine pancreatic cells were transduced for 4 hours on day 1 (the day of isolation). After the 4 hours transduction period, EGFP was already weakly detectable in adenovirally transduced cells (Additional file 2: Figure S2), whereas no EGFP was observed in the lentivirally transduced cells on day 1 (not shown). The number of EGFP+ cells and the amount of protein per cell increased with increasing MOI’s (Additional file 2: Figure S2). Lentiviral expression of EGFP reached a plateau only at day 3 (Figure 3G). During the first days following transduction, adenoviral transduction of rat exocrine cells resulted in high efficiencies and a very high level of transgene expression, even at MOI 1, as compared to Le^VSV-G^CMV-EGFP (Figure 3). However, from day 4 on, massive cell death occurred in the adenovirally transduced cells and the formation of monolayers was disturbed (Figure 3C-E). On day 8, only 31.7 ± 5.3% (n=3) of the adenovirally transduced cells survived, while the viability of cells transduced with Le^VSV-G^CMV-EGFP was comparable to that of non-transduced cells and showed normal monolayer formation and stable transgene expression (Figure 3F-J).

### In vivo transduction of pancreatic cells with lentiviral vectors

To investigate the efficiency of in vivo transduction with Le^VSV-G^CMV-EGFP, viral vectors were injected into the pancreatic parenchyma of CD1 mice (n = 5). A range of 0 - 10^7^ transducing units (TU) was used. Injection of the lectin wheat germ agglutinin coupled to tetramethyl rhodamine isothiocyanate (WGA-TRITC) was previously shown to result in specific binding to acinar cells and served as a control for the injection [14]. Pancreata were analysed for lectin labelling and EGFP expression by endogenous fluorescence in cryosections of the pancreas and by immunohistochemistry with anti-green fluorescent protein (GFP) on paraffin sections on day 7 following viral vector injection. Under these conditions pancreas histology remained normal. However, while lectin-bound TRITC was easily detectable, no EGFP could be observed in viral vector-injected pancreata (Additional file 3: Figure S3). Similarly, no EGFP+ cells could be detected 7 days following injection of different amounts of Le^VSV-G^CMV-EGFP in the parenchyme of rat pancreas (n = 9) (between 0 and 4 × 10^7^ TU) (not shown). In conclusion, pancreatic cells could not be transduced in vivo by intra-parenchymal administration of lentiviral vectors.

### In vivo transduction of pancreatic cells by adenoviral vectors

**Figure 4:**
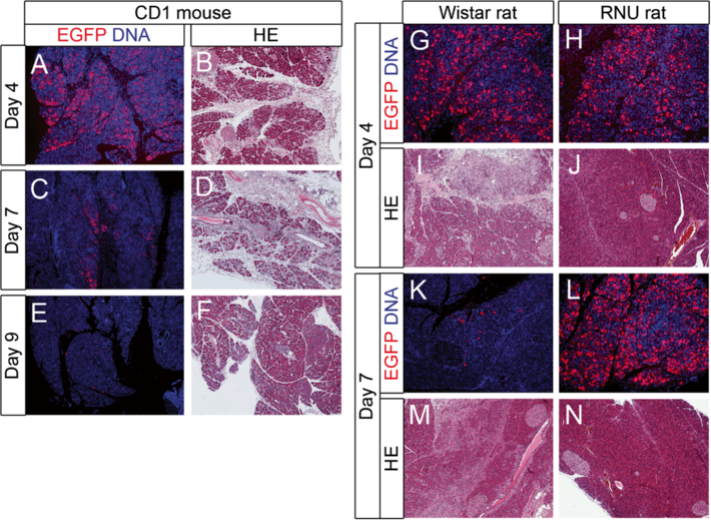
**Intra-parenchymal administration of AdCMV-EGFP in mouse and rat pancreas.** (**A**-**F**) Paraffin sections of the pancreas of CD1 mice injected with (**A, B**) 10^9^ Pfu AdCMV-EGFP analysed on day 4 after injection, (**C-F**) with 2 x 10^9^ Pfu AdCMV-EGFP analysed (**C-D**) on day 7 or (**E-F**) day 9 after injection. (**A, C, E**) Immunohistochemical staining for EGFP in red and DNA in blue. (**B, D, F**) Haematoxylin-eosin staining. (**G-N**) Comparison of intra-parenchymal injection of 5 × 10^9^ Pfu AdCMV-EGFP in (**G, I, K, M**) immune-competent (Wistar) rats and (**H, J, L, N**) immune-deficient (RNU) rats, analysed (**G-J**) on day 4 and (**K-N**) day 7 after injection. (**G-H, K-L**) Immunohistochemical staining for EGFP in red and DNA in blue. (**I-J, M-N**) Haematoxylin-eosin staining.

In contrast with the previous in vitro observations, in vivo administration of adenoviral vectors into the parenchyma of mouse and rat pancreas resulted in high transduction efficiencies. The pancreas of CD1 mice (n = 14) was injected with different amounts of AdCMV-EGFP (0–5 × 10^9^ plaque forming units (Pfu) at multiple sites. Injection of WGA-TRITC was used as a control. Samples were analyzed at day 4 (n = 6), 7 (n = 4), 9 (n = 2) and 12 (n = 2) after injection. On day 4 several lobes of the pancreas showed a high expression level of EGFP (Figure 4A). EGFP was already detectable at a dose of 10^8^ Pfu AdCMV-EGFP and continuously increased as higher amounts of adenoviral vectors were used. However, even at the highest dose used, some pancreatic regions remained negative. The expression of transgene peaked on day 4 (Figure 4A) and diminished with time (Figure 4 A,C,E) to reach zero level at day 12 (not shown). Haematoxylin-eosin stainings showed local inflammation and infiltration of leukocytes on day 4 with a gradual restoration of normal pancreas histology over time (Figure 4B,D,F). Leukocyte infiltration could no longer be detected at day 12 after transduction (not shown).

**Figure 5:**
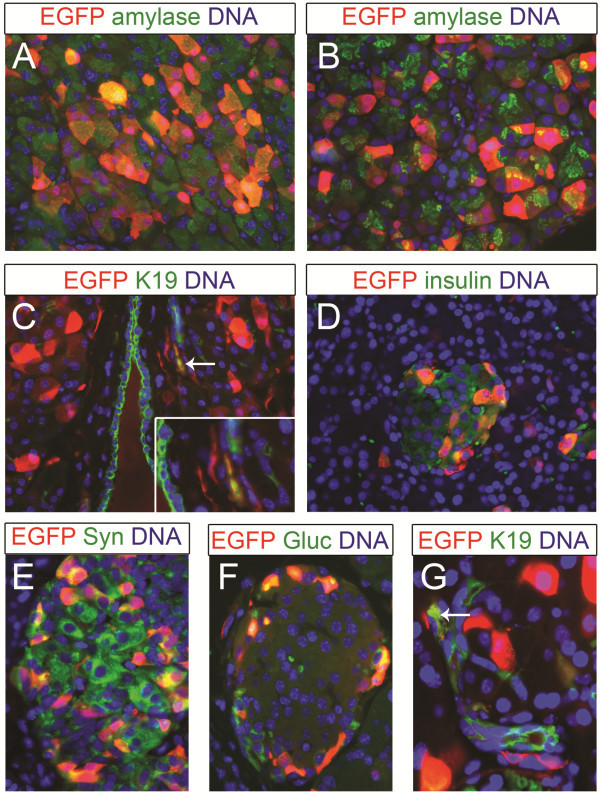
**Double immunohistochemical stainings on mouse and rat pancreas injected intra-parenchymal with AdCMV-EGFP.** (**A**-**G**) Immunohistochemical staining in red for EGFP and in green (**A-B**) for amylase, (**C, E**) keratin 19, (**D**) insulin, (**F**) glucagon, (**G**) synaptophysin. (**A, C, E-F**) Mouse pancreas injected with 10^9^ Pfu AdCMV-EGFP. (**B, D**) RNU rat injected with 5 × 10^9^ Pfu AdCMV-EGFP. (**G**) Wistar rat injected with 5 × 10^9^ Pfu AdCMV-EGFP. Arrows indicate double positive cells for EGFP and keratin 19. The inset in C is a higher magnification.

AdCMV-EGFP (10^9^–5 × 10^9^ Pfu in a volume of 200 – 600 μl of physiologic fluid) were injected at multiple sites in the parenchyma of Wistar rat pancreas. Analysis was done at days 3 (n = 11), 6 (n = 6), 7 (n = 3), 8 (n = 2), and 10 (n = 2). Immunohistochemical detection of EGFP revealed that the higher the volume, the more epithelial cells were transduced (not shown). At a constant number of viral vectors (5 × 10^9^ Pfu) in a small injection volume (200 μl), mesenchymal cells between pancreatic lobes and peripherally located epithelial cells were transduced, whereas with a higher volume (≥ 400 μl) a high transduction efficiency was observed in the centre of the pancreatic lobes (not shown). Also, with increasing numbers of adenoviral vectors, increasing numbers of EGFP+ cells were detected, similar to the results seen in mice (not shown). However, some regions remained negative for the transgene as well. As in mice, the reporter expression was maximal on day 3–4 (Figure 4G and not shown) but declined over time more rapidly than in mice (Figure 4G,K) and was completely absent by day 10 (not shown). Clear signs of local inflammation and leukocyte infiltration were present on day 3–4 and gradually decreased with time (Figure 4I,M). Analysis of consecutive sections showed a clear overlap of inflammatory regions and regions expressing EGFP, as expected (not shown). Double immunohistochemical staining for EGFP and amylase revealed that the majority of the transduced cells both in mice and rats are acinar cells. However, also synaptophysin, insulin or glucagon positive islet cells and some keratin 19-positive duct cells were transduced, albeit at a lower efficiency (Figure 5). In summary, injection of adenoviral vectors into the parenchyma of rodent pancreas resulted in high transduction efficiency but was accompanied by inflammation, and transgene expression was transient.

### In vivo transduction of pancreatic cells from immune-deficient rodents with adenoviral vectors

In order to further improve in vivo transduction of the pancreas, a preliminary study was conducted to compare immune-deficient with immune-competent rodents. Immune-deficient BALB/c Nu/Nu mice (n = 4) were transduced with 2 × 10^9^ Pfu of AdCMV-EGFP as described above and were compared to wild-type BALB/c mice (n = 4). Analysis was done at day 4 and at day 7 after injection. The preliminary results indicate that the effects of in vivo pancreas transduction in BALB/c mice were similar as in CD1 mice, with a high level of reporter expression seen on day 4 and less on day 7, while areas of the transduced pancreas contained inflammatory cells at both time points. Expression of the transgene in BALB/c Nu/Nu mice on day 4 was comparable with the immune-competent mice. However, at day 7 the expression remained high in the immune-deficient mice and less inflammation was present (not shown). Also in rats a preliminary comparative study was performed of in vivo adenoviral pancreas transduction in immune-competent (Wistar) (n = 4) and immune-deficient (RNU/RNU) animals (n = 6). All animals received 5 x 10^9^ Pfu. Two animals from each group were analyzed on day 4, 7 and 14 after injection. The immune-deficient nude rats responded in a similar way as the immune-deficient BALB/c Nu/Nu mice. On day 4 after transduction there was no difference in the expression of the reporter between immune-deficient rats and immune-competent rats, but pancreata of nude rats contained much less inflammation (Figure 4G-J). On day 7, EGFP expression was low in Wistar rats, whereas it remained high in nude rats (Figure 4K-N). In nude rats the structure of the pancreas looked normal by day 7 in contrast to Wistar rats (Figure 4M-N). Even after 14 days, expression of the transgene remained high in the nude rats, in contrast to immune-competent animals (not shown). In conclusion, pancreatic cells could be efficiently transduced in vivo by intra-parenchymal administration of adenoviral vectors in rat and mouse pancreas. Highest efficiency was seen in acinar cells, and pancreatic histology was severely affected in normal pancreas but preliminary results indicated nearly normal pancreas histology in immune-deficient animals.

## Discussion

In the search for methods for efficient gene delivery into pancreatic acinar cells, we have investigated different viral and non-viral vectors in vitro and in vivo. Non-viral in vitro transfection of primary exocrine pancreatic cells only reached very low efficiency. Lipofectamine 2000, widely used for transfection of cell lines, has, to our knowledge, not been reported before for transfection of exocrine pancreatic cells. Many studies indicate very high transfection efficiency in rapidly dividing cells [13,38-41]; while in non-dividing human pancreatic islets lipofectamine 2000 resulted in low transfection efficiency [42]. We have demonstrated previously that the exocrine cells in our cultures show a very limited proliferative capacity [12,43], a possible explanation for the observed low transfection efficiency. Another study indicated that trypsin-dispersion of human, rodent and foetal porcine islets increased the efficiency of gene transfer with lipofectamine 2000 [44]. Our unpublished results showed that enzymatic and non-enzymatic dispersion of exocrine cell clusters had a dramatic negative effect on cell survival. The efficiency of transfections with the non-liposomal lipid effectene in primary cells is dependent on the cell type: some primary cell types were reported to express high amounts of the transgene [45-47] while others showed a low efficiency of transfection [48] comparable to our results.

In general, viral vectors are more efficient than non-viral vectors for gene transfer. One way to alter the tropism of a viral vector is pseudotyping, i.e. the incorporation of heterologous envelope proteins in the vector. The most widely used envelope for lentiviral vectors is VSV-G, as it provides the vectors with a very broad host range, and the viral particles can easily be concentrated by ultracentrifugation. For the in vitro transduction of rat exocrine pancreatic cells, we also tested Mokola pseudotyped lentiviral vectors. Mokola is a rabies-related virus and belongs to the family of Rhabdoviridae, as vesicular stomatitis virus does. Mokola pseudotyped lentiviral vectors have been used for the transduction of a variety of cell types, especially brain cells such as neurons, oligodendrocytes, and astrocytes [49-51] but also skeletal and cardiac myocytes [52], satellite cells [53], skin fibroblasts [54], retinal pigment epithelium [55-57] and alveolar epithelium [58]. With the exception of the study of Kobinger et al. [31], in which only a very limited expression of the transgene was shown in human islet cells, they have not been used for the transduction of pancreatic cells. VSV-G pseudotyped lentiviral vectors have also been used for the delivery of genes into various cell types in vitro, including human and rodent pancreatic islets [29,30,32,33]. However, they have not been used for the transduction of pancreatic acinar cells in vitro. We report that VSV-G pseudotyped lentiviral vectors have an 8–11 times higher efficiency to transduce rat exocrine acinar pancreatic cells compared to Mokola pseudotyped lentiviral vectors at comparable MOI. The transgene expression reached a plateau at 48 hours following transduction, remained stable throughout the culture period and did not affect the viability of the cells. Adenoviral vectors, on the other hand, have been reported to induce a very early and titer-dependent expression of the transgene in pancreatic acinar cells in vitro with a very high expression level after 24 hours [15,21-23,25,28], which is comparable to what we observed. Therefore, for short-term culture, adenoviral vectors are very efficient in gene transfer. However, later in culture they induced a considerable amount of cell death. This has not been reported in the abovementioned studies, since the acinar cells were mostly kept in culture only for 24 hours [21-23] and at maximum for 4 days [15].

The results of in vivo intra-parenchymal delivery of lentiviral vectors in the pancreas are in sharp contrast with the high efficiency during in vitro lentiviral transduction. A similar report has been published, in which no significant transduction of the pancreas by intra-parenchymal injection of lentiviral vectors was reported [35]. On the contrary, intra-ductal administration of lentiviral vectors in the mouse pancreas efficiently transduced acinar cells [34]. In vivo transduction efficiencies do not always correlate with in vitro efficiencies [27,35,59]. The remarkable difference in transduction efficiency of lentiviral vectors in vitro and in vivo might be explained by lentiviral instability in the presence of serum [35,60-63]. Lentiviral vectors also have a relatively large virion size which could affect their capacity to perfuse the tissue [35].

Adenoviral vectors have been used previously to transduce pancreatic cells in vivo via different routes: intravenously [24,26], intra-ductally [64], and intra-parenchymal [17,27]. Although adenoviral vectors show a high efficiency of transduction, they also elicit an immune response. Our preliminary results in immune-deficient nude mice and rats indicated considerably less tissue inflammation. Similar observations have been reported with Rag −/− immune-deficient mice [17].

Several mechanisms may account for the transient transgene expression following adenoviral transduction, both in vivo and in vitro. The adenoviral episome may be eliminated in cells that are replicating, since it does not integrate into the host genome. Furthermore, acute direct toxicity of adenoviral proteins has been described [65,66]. Also, some adenoviral gene products induce apoptosis [67]. Residual expression of adenoviral genes in islet cells in vitro was sufficient to modulate cell death [68]. Moreover, it has been reported that the transgene itself also can induce increased cell death [66,69]. However, in the current study the latter is very unlikely since lentiviral overexpression of EGFP did not induce cell death. The presence of inflammatory infiltrates in the pancreas after intra-parenchymal delivery of adenoviral vectors and the increased duration of transgene expression in immune-deficient animals indicate that the elicited immune response limited the transgene expression.

## Conclusions

We demonstrated that VSV-G pseudotyped lentiviral vectors provide the best transduction efficiency with optimal cell viability for in vitro gene delivery to exocrine pancreatic cells as compared to adenoviral vectors, Mokola pseudotyped lentiviral vectors and lipofection. Remarkably, direct intrapancreatic injection of lentiviral vectors in rodents was highly inefficient. For in vivo transduction of rodent pancreas, adenoviral vectors were superior and our preliminary results in immune-deficient animals were very promising . Mainly exocrine acinar cells were targeted. Acinar cells represent an interesting cell type to study in the context of pancreatic pathology and regenerative biology, since their transdifferentiation potential has already been extensively documented [4-11,13-17,19,20]. The gene transfer methods that we have developed in this study will be helpful not only to allow genetic lineage tracing but also for gain- and loss-of function studies in order to obtain a better understanding of acinar cell plasticity.

## Methods

### Animals

The following animals were used for the in vivo administration of viral vectors: male CD1 mice weighing 38–51 g (Crl:CD1(ICR)) (Charles River Laboratories, L’Arbresle Cedex, France), male BALB/c mice weighing 23–25 g (BALB/cAnNCrl) (Charles River Laboratories), male BALB/c Nude Mice weighing 21–24 g (CAnN.Cg-Foxn1nu/Crl) (Charles River Laboratories), male Wistar Han rats weighing 210–350 g (Crl:WI(Han) (Charles River Laboratories), male Rowett nude rats weighing 180–210 g (HsdHan™:RNU-Foxn1rnu (Harlan, Horst, the Netherlands)). Male 10–12 week old Wistar rats (Janvier, Le Genest-St-Isle, France) weighing 250–300 g were used for the isolation of cells from the pancreas. Pancreata were partially dissociated with collagenase and exocrine acini were purified by centrifugal elutriation as published before [12]. All animal experimentation was approved by the Ethical Committee of the Free University of Brussels.

### Culture procedure

After isolation, the cell density of the suspension of rat exocrine cells was determined using a haemocytometer. Therefore, a representative sample of the cell suspension was centrifuged, followed by trypsinisation (Sigma) for 5 minutes at 37°C. The cells were washed with standard medium with 10% serum (see below) and were then lysed and stained by propidium iodide lyse buffer (sodium citrate (1 g/l), propidium iodide (50 mg/l), tritonX (0.1%)). The nuclei were counted in a Bürker chamber. The exocrine cells were pre-cultured for 4 days in bacteriological Petri dishes (Nunc, Langenselbold, Germany) in Advanced RPMI-1640 medium (Invitrogen, Merelbeke, Belgium) supplemented with 10% foetal bovine serum (FBS) (Gibco, Invitrogen), glutamax-I (Gibco, Invitrogen) and penicillin-streptomycin solution (100 U/l - 100 mg/l) (Sigma, St Louis, MO, USA) at 37°C in a humidified atmosphere of 5% CO_2_. Geneticin sulphate (50 μg/ml) (Sigma) was used to suppress fibroblast overgrowth in the culture. Medium was replaced daily during this preculture period. At the end of the pre-culture, cells were transferred to 24-well plates (Falcon, BD Biosciences, Erembodegem, Belgium) to form adherent cultures. Adherent monolayers were further cultured with RPMI supplemented with 1% FBS and antibiotics and 50 ng/ml human recombinant epidermal growth factor (Sigma) [43].

### Transfection

Rat exocrine cells were transfected with pEGFP-N2 on the day of isolation (day 1) using lipofectamine 2000 (Invitrogen) or effectene (Qiagen, Venlo, Netherlands) according to the manufacturer’s protocol in suspension 24-well plates (Greiner Bio-one, Frickenhausen, Germany) or 35 mm suspension dishes (Nunc). After 4 hours transfection with lipofectamine 2000, the medium was changed to the standard medium with antibiotics and the cells were cultured according to the standard protocol.

### In vitro transduction

After isolation, the rat exocrine cells were transduced by adenoviral or lentiviral vectors (day 1) in minimal amounts of medium without serum for 4 hours in suspension 24-well plates (Greiner Bio-one) or 35 mm suspension dishes (Nunc). Different multiplicities of infection were used. Then the cells were washed three times and cultured according to the standard protocol.

### Viral vector production

Recombinant replication-deficient adenoviral vectors expressing EGFP (AdCMV-EGFP) were generated following standard techniques as described by He et al [70]. Therefore the AdEasy^TM^ adenoviral vector system (Agilent technologies, Diegem, Belgium) was used. The adenoviral plasmids were produced by homologous recombination in electro competent *E.coli* BJ5183 cells between the adenoviral backbone plasmid vector, pAdEasy-1, and a shuttle vector pAdTrack. pAdEasy-1 contains most sequences from human adenovirus serotype 5 with deletion of the genes E1 and E3; the pAdTrack vector is a shuttle vector for production of EGFP-trackable viral vectors. The adenoviral vectors were then produced by transfection in 293E1 cells [70].

The lentiviral vectors used are third generation lentiviral vectors with as main features: the vectors are self inactivating (SIN) (promoter activity in the U3 region of the long terminal repeat (LTR) has been deleted (DeltaU3)) and they contain the DNA flap region also named Triplex or central purine pyrimidine track (cPPT) that is crucial for optimal transport of the reverse transcribed dsDNA into the nucleus [71]. Recombinant lentiviral vectors (LeCMV-EGFP) were produced by transient transfection of 293T cells according to standard protocols [72] with some modifications. Briefly, 293T cells were cotransfected with the packaging plasmid pCMVdeltaR8.74, the envelope plasmid pMD2.G encoding for VSV-G or Mokola envelope, and the transfer vector pTrip-CMV-EGFP-deltaU3 [73]. After 6–8 h the medium was changed and lentiviral vectors were harvested 48 h and 72 h later. Supernatants were treated with DNase (Roche, Vilvoorde, Belgium), filtered through a 0.22-μm-pore-size filter, and concentrated by ultracentrifugation in a Beckman SW28 rotor (Optima LE-80K ultracentrifuge; Beckman Coulter, Palo Alto, CA) for 90 min at 22 000 rpm. After ultracentrifugation, the pellet was resuspended in phosphate buffered saline, divided into aliquots and frozen at −80°C until use. Lentiviral vectors were titrated as described [74].

### Microscopy and immunohistochemistry

Microscopic images were acquired with a Nikon TE2000-E microscope using NIS AR2.30 Imaging Software (Nikon France SAS, Champigny-sur-Marne, France) and with a Leica DM IRBE microscope using Axiovision 3.1 software (Carl Zeiss, Zaventem, Belgium). Pictures were taken from living cell cultures as well as from fixed, stained material.

For in vitro culture, the cells were fixed on day 8 with formaldehyde (Labonord, Templemars, France) for 10 min at room temperature. The monolayer was covered with Vectashield containing 4^′^,6-diamidino-2-phenylindole (Vector Laboratories, Burlingame CA94010, USA). To determine the efficiency of transfection/transduction, the native fluorescence of EGFP of at least 1000 cells per condition was evaluated.

For immunohistochemistry, the pancreata were fixed with formaldehyde (Labonord, Templemars, France) for 4 h, dehydrated and embedded in paraffin. Paraffin sections of 4 μm were cut. Alternatively, the pancreata were fixed for 4 hours in cold 4% paraformaldehyde (Sigma), soaked overnight in 20% sucrose solution, embedded in optimal cutting temperature compound (Labonord) and frozen in liquid nitrogen. Frozen sections of 5 μm were cut. The indirect method with fluorochrome-labelled secondary antibodies was used. Secondary antibodies directly coupled to tetramethyl rhodamine isothiocyanate, fluoresceinisothiocyanate or cyanine 2 were purchased from Jackson ImmunoResearch Laboratories (West Grove, PA., USA). The primary antibodies were: 1/500 rabbit anti-amylase (Sigma), 1/100 goat anti-GFP (Abcam, Cambridge, UK), 1/3000 rabbit anti-glucagon (Prof. C. Van Schravendijk, Brussels), 1/2000 mouse anti-glucagon (Sigma), 1/3000 guinea pig anti-insulin (Prof. C. Van Schravendijk, Brussels), 1/100 rat anti-cytokeratin 19 (Hybridoma bank), 1/1000 rabbit polyclonal anti-keratin (Dako, Glostrup, Denmark) and 1/10 rabbit anti-synaptophysin (Novocastra, Zaventem, Belgium).

### In vivo transduction

Mice were anesthetized by i.p. injection with a mixture of ketamine (75 mg/kg) and medetomidine (1 mg/kg), rats by i.p injection of Nembutal. Exposure of pancreas was performed via laparotomy through midline incision. The lentiviral and adenoviral vector solutions were microinjected directly into the pancreatic parenchyma at multiple sites. In mouse pancreas, we injected in both head and tail part of the pancreas; in rat pancreas, only injections in the tail part were performed. Injections with wheat germ agglutinin, coupled to tetramethyl rhodamine isothiocyanate (Invitrogen) were used as a control [14].

## Abbreviations

AAV: Adeno-associated viral vectors; AdCMV-EGFP: Adenoviral vector carrying CMV-EGFP; CMV: Cytomegalovirus promoter; cPPT: Central purine pyrimidine track; EGFP: Enhanced green fluorescent protein; FBS: Foetal bovine serum; GFP: Green fluorescent protein; Le^Mo^CMV-EGFP: Mokola pseudotyped lentiviral vector carrying CMV-EGFP; Le^VSV-G^CMV-EGFP: VSV-G pseudotyped lentiviral vector carrying CMV-EGFP; LTR: Long terminal repeat; MOI: Multiplicity of infection; PanIN: Pancreatic intraepithelial neoplasias; PDAC: Pancreatic ductal adenocarcinomas; Pfu: Plaque forming units; SIN: Self inactivating; TU: Transducing units; VSV-G: Vesicular stomatitis virus-glycoprotein; WGA-TRITC: Wheat germ agglutinin coupled to tetramethyl rhodamine isothiocyanate.

## Competing interests

The authors declare that they have no competing interests.

## Authors’ contributions

All authors participated in the conception and design of the study, in the critical revision of the manuscript for important intellectual content, and all authors read and approved the final manuscript. IH performed the research (acquisition, analysis and interpretation of data), and drafted the manuscript. LBo, LBa and HH participated in the analysis and interpretation of data and LBo helped in drafting the manuscript.

## Supplementary Material

Additional file 1 **Figure S1.** Shows images of rat exocrine pancreas cells after in vitro transfection with effectene on day 2 and day 7 in culture. Click here for file

Additional file 2 **Figure S2.** Shows images of rat exocrine pancreas cells immediately after transduction with AdCMV-EGFP with different MOI’s. Click here for file

Additional file 3 **Figure S3.** Shows immunohistochemistry of mouse pancreas after intra-parenchymal administration of Le^VSV-G^CMV-EGFP with or without lectin. Click here for file
