# Supplementary material for: Gene delivery to pancreatic exocrine cells in vivo and in vitro
Source: BMC Biotechnol. 2012 Oct 22;12:74. doi: 10.1186/1472-6750-12-74 (PMC3487942; doi:10.1186/1472-6750-12-74)
Supplement: Additional file 1 — Figure S1. Shows images of rat exocrine pancreas cells after in vitro transfection with effectene on day 2 and day 7 in culture. [file 1472-6750-12-74-S1.pdf]

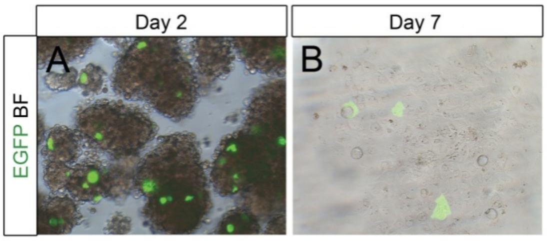

<u>Supplementary Figure 1: Rat exocrine pancreas cells after in vitro transfection with effectene on day 2 and day 7 in culture</u>

(A, B) EGFP expression (green) could be detected already from day 2 in culture (A), and remained at least until day 7 (B). Merged pictures of bright field and green fluorescence.
