# Supplementary material for: Gene delivery to pancreatic exocrine cells in vivo and in vitro
Source: BMC Biotechnol. 2012 Oct 22;12:74. doi: 10.1186/1472-6750-12-74 (PMC3487942; doi:10.1186/1472-6750-12-74)
Supplement: Additional file 2 — Figure S2. Shows images of rat exocrine pancreas cells immediately after transduction with AdCMV-EGFP with different MOI’s. [file 1472-6750-12-74-S2.pdf]

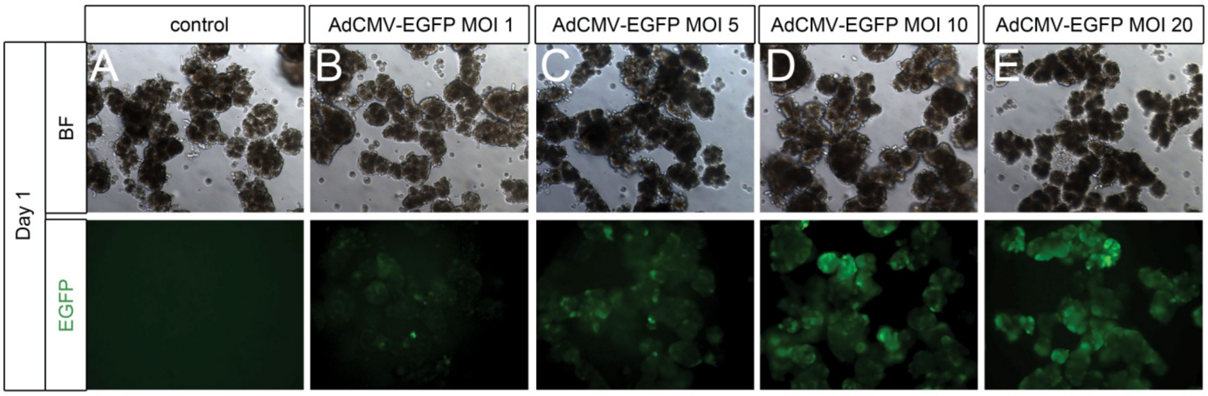

Supplementary Figure 2: Rat exocrine pancreas cells immediately after transduction with AdCMV-EGFP

(A-E) Day 1 in culture of rat exocrine pancreas cells immediately after transduction with AdCMV-EGFP: (A) untransduced, (B) MOI 1, (C) MOI 5, (D) MOI 10, (E) MOI 20. The upper row shows bright field images, the lower row is green fluorescence.
