# Supplementary material for: Gene delivery to pancreatic exocrine cells in vivo and in vitro
Source: BMC Biotechnol. 2012 Oct 22;12:74. doi: 10.1186/1472-6750-12-74 (PMC3487942; doi:10.1186/1472-6750-12-74)
Supplement: Additional file 3 — Figure S3. Shows immunohistochemistry of mouse pancreas after intra-parenchymal administration of LeVSV-GCMV-EGFP with or without lectin. [file 1472-6750-12-74-S3.pdf]

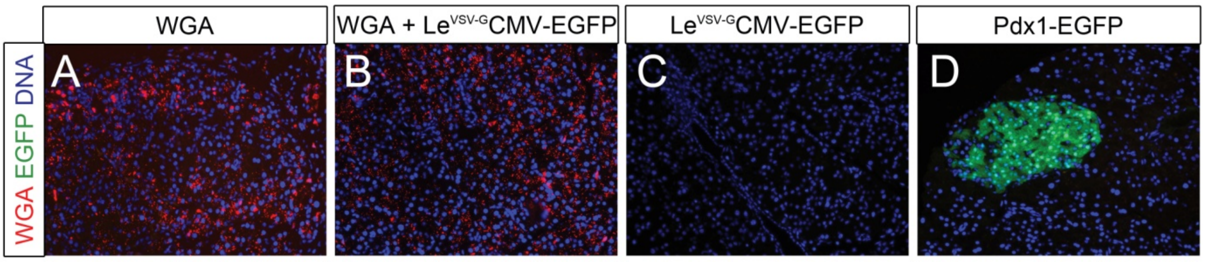

Supplementary Figure 3: Intra-parenchymal administration of Le^{VSV-G}CMV-EGFP with or without lectin in mouse pancreas

(A-C) Cryosections of the pancreas of CD1 mice injected into the parenchymal tissue with (A) WGA-TRITC, (B) mixture of WGA-TRITC and 10^7 TU Le^{VSV-G}CMV-EGFP or (C) 10^7 TU Le^{VSV-G}CMV-EGFP. (D) Pancreas of Pdx1-EGFP served as a control for the detection of EGFP. The lectin was detectable (A-B), whereas no EGFP could be detected in pancreas injected with lentiviral vectors (B-C).
